# Beyond the hot flashes: how machine learning is uncovering the complexity of menopause-related depression

**DOI:** 10.1017/S1092852924002463

**Published:** 2025-01-06

**Authors:** Graziella Orrù, Rebecca Ciacchini, Anna Conversano, Ciro Conversano, Angelo Gemignani

**Affiliations:** Department of Surgical, Medical and Molecular Pathology and Critical Care Medicine, University of Pisa, Pisa, Italy

**Keywords:** menopause, perimenopause, machine learning, psychology, depression, psychological assessment in menopause

## Abstract

**Background:**

The transition into menopause marks a significant stage in a woman’s life, indicating the end of reproductive capability. This period, encompassing perimenopause and menopause, is characterized by declining levels of estrogen and progesterone, leading to various symptoms such as hot flashes, sleep disturbances, sexual dysfunction, and mood irregularities. Moreover, cognitive functions, notably memory, may decline during this phase.

**Objective:**

This exploratory study aimed to evaluate psychological factors in a sample of 98 women recruited from a diagnostic-assistance hospital pathway (AOUP).

**Methods:**

Psychological variables, including depression, anxiety, stress, sleep quality, memory, personality traits, and mindfulness, were assessed using psychometric questionnaires. Machine learning techniques were employed to identify independent variables strongly correlated with higher levels of depression measured by BDI-II.

**Results:**

The findings revealed positive associations between depression and anxiety, stress, low mood, poor sleep quality, and memory complaints, while mindfulness showed a negative correlation. Remarkably, the machine learning analysis achieved a high classification accuracy in distinguishing between individuals with different levels of depression (low vs high).

**Conclusions:**

These results underscore the importance of addressing psychological factors during menopause and offer valuable insights for future research and the development of targeted clinical interventions aimed at enhancing mental health and quality of life for women during this transitional phase.

## 1. Introduction

### 1.1. Menopause and psychological well-being

Menopause (MP) refers to the permanent cessation of menstruation due to the decline of ovarian follicular function, typically diagnosed after 12 consecutive months of amenorrhea.^1^ Except for cases where MP is induced by surgical procedures, it occurs gradually as ovarian function declines, resulting in a progressive and irregular decrease in estrogen and progesterone secretion.^2^ The typical onset of MP generally occurs between the ages of 45 and 55, although individual variability exists.^1^ ^,^ ^3^ ^,^ ^4^ In Italy, the average age for menopausal transition ranges from 50.9 to 51.2 years.^5^ ^,^ ^6^

Scientific research has outlined the stages of MP, offering insights into the physiological changes occurring during this period.^7^ This stage-based approach facilitates the identification and management of symptoms and conditions associated with MP. It comprises three main phases: perimenopause, MP, and post-menopause.

Perimenopause marks the transition towards MP; during this phase, hormonal levels, particularly estrogen and progesterone, may fluctuate significantly. Irregular menstrual cycles often mark one of the initial indicators of perimenopause, with periods becoming unpredictable. Additionally, hot flashes are common occurrences, manifesting during both daytime and nighttime and potentially disrupting sleep, leading to fatigue and irritability.^8^ It is important to note that perimenopausal experiences differ widely; while some women may experience minimal discomfort, others may find this phase more challenging.^9^ ^–^^12^ Throughout perimenopause and MP, circulating levels of estrogens and progesterone decline, causing alteration of brain functions, including mood and sleep changes.^13^ Moreover, such hormonal fluctuations may adversely affect cognitive functioning, heightening the risk of memory problems and cognitive decline. As highlighted by Toffol and colleagues,^14^ estrogen and progesterone receptors are widely distributed in several areas of the adult brain, with estrogen receptors (ERs) found not only in the hypothalamus but also in the hippocampus and frontal lobes (which are involved in supporting verbal memory, working memory, and retrieval), amygdala, locus coeruleus, cerebellum, pituitary, basal forebrain, cerebral cortex, and glial cells. Additionally, several types of neurons (cholinergic, noradrenergic, serotoninergic, and dopaminergic) respond to estrogens and progesterone. Estrogens may play a crucial protective role against the typical aging-related decline in these cognitive abilities.^15^

Post-menopause is the phase that follows MP, marked by the permanent cessation of menstruation and fertility.^16^ Many symptoms associated with MP, such as hot flashes and night sweats, tend to decrease as the body adjusts to hormonal changes, gradually reducing initial imbalances.^17^ However, some women may still experience symptoms. Notably, post-menopause involves long-term changes in the body due to the persistent lack of estrogen, which can increase the risk of osteoporosis and cardiovascular issues. Furthermore, post-menopause may result in changes in weight and body fat distribution.

Despite being a natural process, many women find MP to be a challenging period. Hormone replacement therapy (HRT) is a conventional treatment option for menopausal symptoms, but it is not risk-free.^18^ Other non-hormonal medications, such as antidepressants and lifestyle adjustments can be beneficial in managing menopausal symptoms.

This study aims to evaluate various psychological aspects in a sample of menopausal women participating in a cardiovascular risk management program at the *Azienda Ospedaliero Universitaria Pisana* (AOUP). The aims encompass assessing levels of depression, anxiety and stress, sleep quality, prospective and retrospective memory complaints, personality factors, and mindfulness ability using various psychological assessment tools in a cross-sectional examination.

## 2. Materials and methods

### 2.1. Participants, inclusion and exclusion criteria

One hundred and eighteen Italian-speaking women were recruited for the present study. Of these, 20 women were excluded due to the lack of a comprehensive evaluation, resulting in a final sample of n = 98 (mean age = 57.20 ± 4.50 years).

Inclusion criteria for the study were as follows: (1) menopausal women; (2) age range between 45 and 65 years; (3) adequate understanding of the Italian language; and (4) the ability to provide informed consent. Exclusion criteria included: (1) conditions other than menopause (e.g., perimenopause); (2) ages outside the specified range; (3) insufficient comprehension of the Italian language; and (4) inability to provide informed consent.

### 2.2. Assessment

The participants underwent evaluation by an experienced psychologist from the Clinical Psychology Unit at Santa Chiara Hospital (Pisa, Italy), with each assessment lasting approximately one hour per participant. The psychological assessment included the following tests in their Italian version:

- The *Beck’s Depression Inventory* (BDI-II)^19^ ^,^ ^20^: the instrument was developed by Beck, Steer, and Brown.^19^ The BDI-II consists of 21 items, each representing a specific statement related to potential depressive symptoms, such as “*Feeling of sadness*” or “*Loss of interest in pleasurable activities.*” The questionnaire demonstrates a high level of reliability and validity, indicating that it is a consistent tool for measuring depression and providing an accurate assessment of depressive symptoms.
- The *Perceived Stress Scale* (PSS)^21^ ^,^ ^22^: is a 10-item questionnaire widely used to assess stress levels and is a self-assessment tool. The scale has been extensively employed in numerous scientific studies and mental health research to examine the relationship between perceived stress and physical and psychological conditions.
- The *State–Trait Anxiety Inventory* (STAI-Y1 and STAI-Y2)^23^ ^,^ ^24^: is a questionnaire designed to assess anxiety across two dimensions, state-like anxiety (STAI-Y1) and trait-like anxiety (STAI-Y2). It comprises 20 items describing various statements related to anxiety, such as “*I feel calm*” or “*I feel restless.*” Both versions of the STAI scale have been widely used in clinical and research settings to evaluate perceived anxiety as both a transient experience and a stable personality trait.
- The *Pittsburgh Sleep Quality Index* (PSQI)^25^ ^,^ ^26^: is a tool used to assess sleep quality and to identify potential sleep disturbances in adults. PSQI is a self-report questionnaire covering various dimensions of sleep, enabling a comprehensive evaluation of an individual’s overall sleep quality.
- The *Mindful Attention Awareness Scale* (MAAS): is a self-assessment tool developed in 2003^27^ ^,^ ^28^ to measure an individual’s level of mindfulness and mindful attention in everyday life. The scale is designed to assess a person’s propensity to be present and aware of their internal and external experiences without judgment or reactivity.
- The *Prospective and Retrospective Memory Questionnaire* (PRMQ)^29^: is a self-report tool used to assess prospective and retrospective memory in adults. Prospective memory refers to the ability to remember and perform planned activities in the future, whereas retrospective memory involves the ability to recollect past events and information. The PRMQ is widely employed in various research and clinical settings to assess prospective and retrospective memory difficulties in adults.
- The *Big Five Inventory* (BFI)^30^ ^,^ ^31^: is a psychometric tool used to assess personality traits based on the “*Big Five*” model. BFI provides a concise measurement of the main personality traits and provides useful information regarding an individual’s disposition.

### 2.3. Experimental procedures

Before administering the psychological scales and/or questionnaires, an anamnesis was conducted in the following domains: (i) year of onset of the perimenopausal period; (ii) year of onset of menopause; and (iii) lifestyle factors, such as physical activity. The questionnaires were administered in the following sequence: BDI-II, PSS, STAI-Y1, and Y2, PSQI, MASS, PMRQ, and BFI. All participants provided their informed consent.

#### Data analysis

Statistical analyses were performed using Jamovi^32^ and Weka 3.8.6.^33^ To investigate the independent variables associated with BDI, we divided the final sample (N = 98) into three subgroups based on depression levels: low (group: LD), medium (group: MD), and high (group: HD), as measured by BDI-II. For the LD group, participants whose BDI-II scores fell within the 33rd percentile, ranging from 0 to 4 (n = 35; LD group) were retained. Likewise, for the HD group, participants whose scores were above the 66th percentile, ranging from 9 to 28 (n = 34; HD group) were included. Participants with scores closer to the median score (BDI-II score range = 5–8) were computed within the MD group (n = 29, MD group). Next, ML analysis was performed in order to evaluate the classification accuracy between the three groups LD vs MD vs HD.

To reduce bias and prevent overfitting, the k-fold cross-validation method was employed. This involves randomly dividing the dataset into k folds of equal size, where in our case, k was set to 10. Classification quality was assessed using two metrics: F1-score (F1) and AUC-ROC (AUC). The F1 score ranged from 0 to 1, with 1 being the highest and indicating perfect precision and recall. An F1-score of 0 indicates that the classifier made no correct positive predictions. The AUC reflects how well the classifier can distinguish between positive and negative classes. An AUC of 1 indicates perfect accuracy, while an AUC of 0 suggests that the classifier incorrectly predicted all positives as negatives, and vice versa.

## 3. Results

Means and standard deviations for age, body mass index (BMI), and questionnaire scores are reported in Table 1.

**Table 1.** Demographic characteristics and questionnaire scores (n = 98)

	N	Mean	Median	Mode	SD	Range	Min	Max
Age	98	57.20	57.00	57.00	4.50	21	44	65
BMI	98	24.83	24.20	22.00^a^	4.03	19.8	17.6	37.4
BDI-II	98	7.58	7.00	0.00	6.58	28	0	28
PSS	98	16.72	17.00	15.00^a^	6.60	33	1	34
STAI-Y1	98	32.73	30.50	30.00	9.25	55	3	58
STAI-Y2	98	39.16	39.50	40.00^a^	8.57	39	23	62
PSQI	98	6.18	5.00	3.00	3.89	17	0	17
MAAS	98	71.96	72.00	71.00	9.91	46	42	88
PRMQ	98	36.67	34.00	32.00	12.32		16	76
BFI_E	98	27.42	28.00	33.00	5.70	27	12	39
BFI_A	98	34.83	35.00	35.00	4.79	24	21	45
BFI_C	98	36.67	37.00	35.00^a^	5.48	29	16	45
BFI_N	98	22.83	22.00	20.00	6.10	27	9	36
BFI_O	98	37.51	38.00	41.00	6.71	30	20	50

a In the case of more than one mode, only the first is indicated.

Notes: BMI, Body Mass Index; PSS, Perceived Stress Scale; BDI-II, Beck Depression Inventory-ΙΙ; STAI-Y1 e STAI-Y2, State–Trait Anxiety Inventory; PSQI, Pittsburgh Sleep Quality Index; MAAS, Mindful Attention Awareness Scale; PRMQ, Prospective and Retrospective Memory Questionnaire; BFI, Big Five Inventory.

PRMQ, N= 98; Mean = 36.67; Median= 34.00; Mode=32.00; SD= 12.32; Min= 16; Max = 76

### 3.1. Cronbach’s alpha coefficient analysis for scale reliability

To assess the reliability of each scale used in this study, Cronbach’s alpha coefficients were calculated for each scale. Cronbach’s alpha is a commonly used metric to evaluate internal consistency, which reflects how closely related a set of items are within a scale. The calculation of Cronbach’s alpha for each scale involved analyzing the covariance among items within each scale to determine how consistently participants responded to items intended to measure the same construct. A higher Cronbach’s alpha (above 0.7) typically indicates good internal consistency, while values closer to 1 suggest excellent reliability. The specific alpha values for each scale are presented below, allowing for an assessment of each scale’s reliability within the context of this study.

The reliability of each scale administered in this study was assessed using Cronbach’s alpha to evaluate internal consistency. The BDI-II demonstrated good reliability (α = 0.86), suggesting that it consistently measures depressive symptoms among participants. The PSS showed acceptable reliability (α = 0.75), indicating that it is reasonably consistent in assessing perceived stress. The STAY-Y1 (α = 0.89) and STAY-Y2 (α = 0.82) achieved high reliability, indicating that both scales reliably assess different aspects of state and trait anxiety. The MAAS also demonstrated strong reliability (α = 0.82), showing good consistency in measuring mindfulness. The PMRQ yielded an excellent alpha value (α = 0.93), indicating a very high level of internal consistency for assessing personal motivational regulation. In contrast, the BFI displayed questionable reliability (α = 0.67), suggesting that it may have lower consistency in measuring personality traits across items. The PSQI had a notably low alpha value (α = 0.47), which may indicate inconsistency in responses related to sleep quality; this could reflect variability in how participants interpret or respond to items within the PSQI.

Overall, these results suggest that most scales in this study exhibited adequate to excellent reliability, with the PSQI and BFI showing lower levels of internal consistency, which may impact the interpretation of their results.

### 3.2. Correlations

A positive correlation emerged between STAI-Y2 (state anxiety) and PSS (rs = 0.531, p < 0.001), as well as between STAI-Y1 (trait anxiety) and PSS (rs = 0.449, p < 0.001). This suggests that elevated levels of state anxiety are associated with heightened levels of perceived stress and trait anxiety.

The results of the correlation analysis are shown in Table 2. Among the variables considered, a positive correlation was observed between STAI-Y1 (trait anxiety) and PSS (rs = 0.304, p < 0.01), indicating that higher levels of trait anxiety are associated with increased levels of stress. Furthermore, another positive correlation emerged between STAI-Y2 (state anxiety) and PSS (rs = 0.531, p < 0.001), as well as between STAI-Y1 (trait anxiety) and PSS (rs = 0.449, p < 0.001), suggesting that elevated levels of state anxiety are associated with heightened levels of perceived stress and trait anxiety.

**Table 2.** Correlations matrix

		BMI	PSS	STAI-Y1	BDI-II	PSQI	MAAS	PMRQ	BFI_E	BFI_C
BDI-II	Spearman’s Rho	0.241^*^								
	p-value	0.017								
STAI Y1	Spearman’s Rho		0.304^**^							
	p-value		0.002							
STAI Y2	Spearman’s Rho		0.531^***^	0.449^***^						
	p-value		< .001	< .001						
PSQI	Spearman’s Rho				0.424^***^					
	p-value				< .001					
MAAS	Spearman’s Rho				−0.398^***^	−0.238^*^				
	p-value				< .001	0.018				
PMRQ	Spearman’s Rho				0.272^**^	0.213^*^	−0.514^***^			
	p-value				0.007	0.036	< .001			
BFI	Spearman’s Rho				−0.262^**^					
	p-value				0.009					
BFI_A	Spearman’s Rho				−0.260^**^			−0.212^*^	0.352^***^	
	p-value				0.010			0.036	< .001	
BFI_C	Spearman’s Rho					−0.239^*^	0.334^***^	−0.469^***^	0.308^**^	
	p-value					0.018	< .001	< .001	0.002	
BFI_N	Spearman’s Rho				0.351^***^	0.300^**^	−0.284^**^	0.244^*^	−0.362^***^	−0.295^**^
	p-value				< .001	0.003	0.005	0.016	< .001	0.003
BFI_O	Spearman’s Rho						0.274^**^		0.275^**^	
	p-value						0.006		0.006	

* p < .05.

** p < .01.

*** p < .001.

Additionally, a positive correlation was also observed between PMRQ and PSQI (rs = 0.213, p < 0.05), while a negative correlation was found between PMRQ and MAAS (rs = −0.514, p < 0.001). This indicates that higher memory complaints are linked to worse sleep quality. Lastly, among negative correlations, MAAS and PSQI (rs = −0.238, p < 0.05) were observed, indicating that higher levels of mindfulness correspond to better sleep quality.

Regarding the personality questionnaire, a positive correlation was observed between BFI_A (agreeableness) and BFI_E (extraversion) (rs = 0.352, p < 0.001), while a negative correlation was found between BFI_A (agreeableness) and PMRQ (rs = −0.212, p < 0.05). This might suggest that higher levels of agreeableness are associated with elevated levels of extraversion and decreased levels of memory complaints. Furthermore, another positive correlation was identified between BFI_C (conscientiousness) and MAAS (rs = 0.334, p < 0.001), as well as between BFI_C and BFI_E (extraversion) (rs = 0.308, p < 0.01). Conversely, negative correlations were found between BFI_C (conscientiousness) and PSQI (rs = −0.239, p < 0.05), as well as between BFI_C and PMRQ (rs = −0.469, p < 0.001). This may suggest that, in our sample, higher conscientiousness is associated with increased awareness and extraversion, leading to better sleep quality and fewer memory complaints.

Moreover, a positive correlation was observed between BFI_N (neuroticism) and PSQI (rs = 0.300, p < 0.01) and PMRQ (rs = 0.244, p < 0.05). Conversely, negative correlations were found between BFI_N (neuroticism) and MAAS (rs = −0.284, p < 0.01), BFI_E (extraversion) (rs = −0.362, p < 0.001), and BFI_C (conscientiousness) (rs = −0.295, p < 0.01). High levels of neuroticism appear to be associated with poorer sleep quality and increased memory complaints regarding one’s memory efficiency. In contrast, high levels of neuroticism are associated with lower levels of mindfulness, extraversion, and conscientiousness. Lastly, a positive correlation emerged between BFI_O (openness) and MAAS (rs = 0.274, p < 0.01) and BFI_E (extraversion) (rs = 0.275, p < 0.01). This indicates that high levels of openness are associated with elevated levels of awareness and extraversion.

#### Correlations between the variables and BDI-II scores

Concerning the BDI-II scores results and the variables considered, the following correlations were observed:

- a positive correlation between BDI-II and BMI (rs = 0.241, p < 0.5), indicating that higher levels of depression are associated with higher BMI;
- a positive correlation between PSQI and BDI-II (rs = 0.424, p < 0.001), suggesting that poorer sleep quality is associated with increased levels of depression;
- a negative correlation between MAAS and BDI-II (rs = − 0.398, p < 0.001), indicating that higher levels of mindfulness are associated with lower levels of depression;
- a positive correlation between PRMQ and BDI-II (rs = 2.72, p < 0.01), indicating that higher memory complaints are associated with higher levels of depression;
- a negative correlation between BFI_E/BFI_A and BDI-II respectively: rs = 2.62, p < 0.01; rs = −0.260, p < 0.01; positive correlation between BFI_N (neuroticism) and BDI-II (rs = 0.351; p < 0.001). This suggests that lower extraversion/agreeableness is associated with higher levels of depression; conversely, higher neuroticism is associated with higher levels of depression.

### 3.3. Machine learning analysis

As previously stated, to investigate the independent variables that strongly correlated with higher levels of depression, as measured by BDI-II, the sample (N = 98) was divided into three subgroups: individuals with low levels of depression (LD), medium levels of depression (MD) and high levels of depression (HD).

The selection of specific classifiers was based on their complementary characteristics, enabling a comprehensive evaluation of our dataset. By comparing models with distinct underlying assumptions: probabilistic (Naïve Bayes), linear (Logistic Regression and Simple Logistic), non-linear (SVM), and ensemble-based (Random Forest) we aimed to assess both linear and complex feature interactions, potentially improving model interpretability and robustness.

#### Machine learning-based classification accuracies of women with high, medium, and low levels of depression

To investigate which independent variables (age, BMI, physical activity), as well as the total score of the administered questionnaires BDI-II, PSS, STAI-Y1, STAI-Y2, PSQI (and sub-components C1-C7), MAAS, PMRQ (and total score, prospective and retrospective memory scores), BFI (and subcomponents E, A, C, N, O) (25 attributes and 679 instances) correlate most with high, medium and low levels of depression, ML analysis was performed. Therefore, in the first preliminary round, all variables mentioned were used, including the BDI-II score. The highest classification accuracy in distinguishing LD vs MD vs HD groups was around 98.97%, achieved by the Simple logistic classifier (AUC = 0.843; d = 1.42; F1 = 0.99). All classifiers and their performances are shown in Table 3.

**Table 3.** Classifiers and performances within the classification of the groups (LD = 35; MD = 29; HD = 34)

Classifier	Accuracy (%)	AUC	F1	Correct classification		
Naïve Bayes	86.73%	0.886 (d = 1.71)	0.870	LD 30/35	MD 23/29	HD 32/34
Logistic Regression	80.61%	0.807 (d = 1.23)	0.807	LD 31/35	MD 20/29	HD 28/34
Simple Logistics	**98.97**%	0.843 (d = 1.42)	0.990	LD 35/35	MD 29/29	HD 33/34
Support Vector Machine	60.20%	0.694 (d = 0.717)	0.607	LD 25/35	MD 13/29	HD 21/34
Random Forest	97.96%	1.00 (d = 3.091)	0.979	LD 35/35	MD 27/29	HD 34/34

The classification process was repeated by removing a prominent predictor of depression from the classification, which was the BDI-II score. Various classifiers were applied, but the accuracy in classifying the three groups did not exceed 48.97% (Naïve Bayes, AUC = 0.590; d = 0.32; F1 = 0.477). This outcome is likely due to the overlap in characteristics between the MD group and the HD and LD groups. For this reason, the MD group was eliminated from the overall sample, with the sample size therefore composed of HD = 34 and LD = 35 individuals.

#### Independent variable analysis based on the correlations as predictors of the two classes

In the classification process, the PART rule^34^ and its cut-offs were used to identify the variables that demonstrated the best performance in classifying participants into the LD and HD groups. PART decision list identified seven rules, which resulted in an accuracy of 62.32% (as shown in Table 4 in the decision matrix). Although PART was not the most accurate classifier, it was the most interpretable in terms of its rules. Indeed, PART does not require global optimization in order to generate accurate rule sets, which is its primary advantage; it utilizes the separate-and-conquer technique, in which it creates a rule, eliminates the instances it covers, and then recursively continues creating rules for the remaining instances until none are left. To create a single rule, a pruned decision tree is constructed for the current set of cases (instances), the leaf with the greatest coverage is converted into a rule, and the tree is discarded.

1. If PSQI_C5 > 1 AND PMRQ_retrospective memory ≤32 AND > 11 AND PSQI_C1 > 1, then the subject is classified as: **HD** (11.0)
2. If PSQI_C7 ≤ 2 AND BFI_E > 29, then the subject is classified as: LD (22.0/2.0)
3. If PMRQ_retrospective memory ≤12, then the subject is classified as: LD (11.0/1.0)
4. If PSQI_C6 ≤ 0 AND PSQI_C5 > 1, then the subject is classified as: **HD** (9.0/1.0)
5. If PSQI _C6 > 0, then the subject is classified as: **HD** (7.0)
6. If STAI-Y1_TotalScore ≤35: **HD** (6.0/1.0)
7. In all the other cases: LD (3.0)

**Table 4.** Confusion matrix for corrected classification (Accuracy = 62.32%; AUC = 0.626; F1 = 0.623; Recall = 0.623; Precision = 0.623)

	HD	LD	Accuracy for corrected classification
HD	**21**	13	62%
LD	13	**22**	63%

The results reported in Table 5 refer to the comparison between HD and LD women.

**Table 5.** Classifiers and performances within the classification of the groups (LD = 35; HD = 34)

Classifier	Accuracy (%)	AUC	F1	Correct classification	
Naïve Bayes	68.12%	0.687 (d = 0.69)	0.678	LD 27/35	HD 20/34
Logistic regression	69.57%	0.660 (d = 0.58)	0.696	LD 24/35	HD 24/34
Simple logistics	66.67%	0.681 (d = 0.67)	0.660	LD 16/35	HD 28/34
Support vector machine	69.57%	0.602 (d = 0.37)	0.695	LD 26/35	HD 22/34
Random forest	66.67%	0.667 (d = 0.61)	0.667	LD 24/35	HD 22/34

The classifiers that yielded the higher accuracy were *logistic regression* and *support vector machine* (SVM) classifiers.

## 4. Discussion

The purpose of this study was to observe, through the administration of psychometric questionnaires, certain psychological variables typically involved in women passing through perimenopause and menopause.

One of the primary objectives was to identify psychological risk factors consistent with the existing literature, including depression, stress, anxiety, poor sleep quality, and any difficulties in memory capacities.^35^ ^,^ ^36^ The results of the analysis provide a substantial foundation for reflection in relation to this objective.

The average age of our sample was 57.20 years (±4.50); this data is consistent with the average onset age of menopause which ranges from 45 to 55 years old.^37^

In our sample, the average Body Mass Index (BMI) was 24.83 (± 4.03); since a BMI between 18.5 and 24.9 is considered normal and a BMI between 25.5 and 29.9 is classified as pre-obesity, our sample falls into both categories. This may suggest a trend of weight gain in women during menopause, a phenomenon that some studies attribute to hormonal changes occurring during perimenopause and which may contribute to increased abdominal obesity, with further implications for physical and psychological health.^38^ Indeed, some studies conclude that hormonal changes during perimenopause substantially contribute to increased abdominal obesity, leading to further physical and psychological morbidity.^39^ ^,^ ^40^ Scientific evidence has demonstrated that estrogen therapy can partly prevent this change in body composition and its associated metabolic consequences,^41^ while other studies advise against this therapy for managing obesity or weight gain.^42^

The average stress levels of the sample were slightly higher compared to normative data (16.72 ± 6.60). Stress can be particularly detrimental for women in menopause due to a combination of physical, psychological, and hormonal factors.^43^ Chronic stress can trigger an inflammatory response in the body, exacerbate menopause-related symptoms, and contribute to weight gain.^44^ ^–^^46^ Moreover, the emotional instability often accompanying menopause can be amplified by stress, leading to anxiety, depression, and sleep disturbances.^47^ ^,^ ^48^ As is widely recognized, elevated levels of anxiety and depression serve as significant risk factors for various psychopathologies.^49^ ^,^ ^50^

The mean sleep quality score of our sample (PSQI; M = 6.18 ± 3.89) indicates overall poor sleep quality. These results are consistent with the fact that sleep disturbances frequently occur during menopause and may vary significantly. The main causes of these disturbances are linked to hormonal changes, particularly the decrease in estrogen levels; symptoms of sleep disturbances during menopause may include insomnia, night sweats, excessive sweating, non-restorative sleep, and frequent awakenings.^51^

### Anxiety and stress

The results in correlations have highlighted a positive correlation between trait anxiety and perceived stress, suggesting that high levels of trait anxiety are associated with higher levels of stress. Similarly, state anxiety was found to be positively correlated with both PSS and trait anxiety, suggesting that generally anxious women also tend to experience spikes in anxiety in response to specific situations. These findings are consistent with existing literature indicating a link between anxiety and perceived stress in menopausal women, suggesting that targeted interventions to manage anxiety could have a positive impact on overall stress levels during this life stage.^47^ Additionally, a slight negative correlation (rs = −0.179, p < 0.05) was found between BMI and STAI Y 2, indicating a correlation between body mass index and trait anxiety. This finding seems to confirm findings from previous studies in the literature, which have observed associations between high levels of trait anxiety and obesity.^52^

### Mindfulness and sleep quality

A negative correlation was observed between levels of mindfulness and sleep quality, suggesting that higher mindfulness is associated with better sleep quality. This supports hypotheses in the literature concerning the significant role of meditation, particularly mindfulness, in promoting good sleep quality.^53^

Mindfulness practice helps in reducing stress and anxiety, commonly recognized as key factors in sleep disturbances.^54^ ^,^ ^55^ By focusing on the present moment and accepting emotions without judgment, individuals can calm their minds and reduce pre-sleep worrying about past or future events.^56^ For menopausal women, nighttime hot flashes can disrupt sleep; mindfulness can help develop greater awareness of bodily sensations, facilitating acceptance of hot flashes without negative reactions and promoting a return to sleep.

Mindfulness can also enhance awareness of sleep habits and hygiene, allowing individuals to identify and address behaviors that may interfere with sleep. Finally, mindfulness practice has shown efficacy in reducing intrusive thoughts and rumination; being mindful of thoughts and concerns without emotional involvement aids in letting go of disturbing thoughts, promoting relaxation before sleep.^57^ ^,^ ^58^

### Memory complaints

Complaints about prospective and retrospective memory efficiency were found to be correlated with poor sleep quality and negatively with levels of mindfulness. This suggests that poor sleep quality and lower mindfulness are associated with greater complaints about one’s memory.^59^ This finding is consistent with many studies, although many of them distinguish the influence that psychotropic medications may have on sleep and memory quality.^60^ ^,^ ^61^ Learning and mnemonic abilities are important protective factors as individuals age.^62^ The correlation between sleep quality, mindfulness, and memory complaints aligns with research indicating that sleep and awareness influence cognitive functioning, suggesting that improving sleep quality may help mitigate memory concerns.^29^ ^,^ ^60^

### Personality factors

Positive correlations were observed in our sample between agreeableness/friendliness and extraversion, and between conscientiousness, mindfulness, and extraversion. Conversely, high levels of neuroticism are associated with poorer sleep quality and greater complaints about memory, corroborating findings from other studies on the topic.^63^ Mindfulness levels have already been shown to negatively correlate with neuroticism and negative affectivity, and positively with conscientiousness and positive affectivity.^63^

Individuals with high levels of neuroticism - or high negative effect - are more susceptible to psychological distress and are less likely to experience psychological well-being, while those with developed mindfulness skills are less susceptible to psychological distress and are more likely to be psychologically adapted.^63^ ^,^ ^64^ As is well-known, the aspect of personality becomes highly significant when correlated with other psychological variables to better elucidate certain characteristics.^65^

Lastly, significant correlations were observed between depression levels and other variables such as BMI, sleep quality, mindfulness, and memory complaints. The correlation between depression, BMI, and neuroticism observed in this study is partially supported by literature, emphasizing how physical and psychological factors may jointly increase vulnerability to depression in menopause. These findings pave the way for further investigation into the mechanisms underlying these associations.^38^

### Machine learning

The present study utilized ML techniques to analyze psychological variables in menopausal women. ML techniques enable a more thorough analysis of data in different clinical populations [i.e.,^66^ ^–^^68^].

The main results of the ML analysis revealed significant associations between various psychological factors and depression levels. Specifically, we found positive correlations between depression and anxiety, stress, low mood, poor sleep quality, and memory complaints, while mindfulness showed a negative correlation. The ML analysis achieved a high classification accuracy in distinguishing between LD, MD, and HD groups, with a classification accuracy of around 98.97%.

In the classification process that followed, the BDI-II scores, a significant predictor of depression, were removed from the analysis. Despite applying various classifiers, the accuracy in classifying the three groups did not exceed 48.97%. The lower accuracy observed can be attributed to the overlapping characteristics among the groups analyzed presenting different levels of depression (medium, high, and low). To address this issue, the group presenting a medium level of depression (MD group) was excluded from the sample.

The independent variable analysis in the classification process utilized the PART rule to identify variables with the best performance in classifying participants into the two groups with low and high levels depression. The PART decision list established seven rules and defined specific thresholds for psychometric testing, resulting in an accuracy of 62.32%. Despite not being the most accurate classifier, PART was the most interpretable due to its rules.

These findings underscore the potential of ML in identifying key psychological factors associated with menopause-related depression, offering valuable insights for future research and the development of targeted clinical interventions aimed at enhancing mental health and quality of life for women during this transitional phase.

### Depression, memory, and sleep quality

The findings of this study highlight how various psychological and personality factors influence depression levels in menopausal women, underscoring the importance of an integrated approach to managing this condition. The associations between sleep quality, mindfulness, and memory with BDI-II scores suggest that targeted interventions aimed at improving sleep quality and enhancing mindfulness awareness may help alleviate depressive symptoms in this population. Furthermore, the links between personality traits, such as neuroticism and extraversion, and depression indicate that personalized interventions focusing on emotional regulation strategies and psychological support could be especially beneficial for women with specific personality profiles. In summary, these results support the integration of psychosocial assessments and tailored interventions as part of a holistic clinical approach to enhance the quality of life and mental well-being of women undergoing menopausal transition.

In summary, our findings regarding the associations between anxiety, stress, and psychological well-being in menopausal women align with the existing literature. Freeman et al.,^69^ identified a similar impact of hormonal changes on mood, supporting our findings on the link between anxiety, stress, and psychological health. The relationship between mindfulness and sleep quality supports the research of Carmody and Baer,^70^ which highlights mindfulness as a beneficial practice for well-being and sleep quality. Additionally, our findings on memory complaints reflect those of Mitchell and colleagues,^71^ who reported cognitive changes during the menopausal transition. In the context of personality traits, our results are consistent with Weber et al.,^72^ which indicates that neuroticism and other personality factors may influence menopausal experience, potentially exacerbating distress. Lastly, the associations we found on depression, BMI, and neuroticism align with the research of Schmidt and Rubinow^73^ on the interplay between physical and psychological factors affecting mood during menopause. Collectively, these findings reinforce existing theoretical connections, suggesting that an integrated approach may be beneficial for menopausal women.

### Limitations and future directions

This research presents several limitations as follows: (1) the sample size is limited (n = 98) and this may potentially restrict the generalizability of the findings; (2) additionally, the sample was recruited from a specific clinical psychology unit, which may not be representative of the general population of menopausal women. Therefore, the findings may not be generalizable to a broader population; (3) the study has a cross-sectional design, which limits the ability to establish causal relationships between variables; (4) an additional limitation of this study is the lack of detailed information on the use of medications, such as antidepressants, sleeping pills, or hormonal treatments by participants, which may influence symptoms related to sleep, anxiety, stress, and depression. The absence of this data may impact the interpretation of our findings, as these treatments could affect the psychological variables assessed. Future research should incorporate information on medication use to provide a more comprehensive understanding of the factors influencing menopausal symptoms; (5) our reliance on self-report measures for assessing psychological variables introduces potential response biases, which could impact the accuracy of the findings. Implementing objective measures or adopting multi-method approaches could enhance the validity of future studies by reducing such potential biases.

A longitudinal design would provide more robust evidence regarding the relationships observed. Furthermore, the inclusion of a control group could offer a comparison to assess whether the associations identified between variables are specific to menopausal women or whether similar patterns are present in other demographic groups.

Future research should aim to address these limitations by employing longitudinal designs and integrating both objective and self-report measures. Such enhancements would provide a more comprehensive understanding and increase the robustness of findings within this field.

## 5. Conclusions

In summary, the findings of the present research provide preliminary insights into the complex interplay among psychological, behavioral, and personality variables in the context of menopause. While these findings may provide important avenues for further exploration, we recognize that they are preliminary and should be interpreted with caution due to the limitations of the study, including the modest sample size, cross-sectional design, and recruitment from a single clinical setting. Our study primarily aimed to identify preliminary trends and relationships rather than to establish robust generalizability.

Future research with larger, more diverse samples and longitudinal designs would be essential in order to build upon these initial findings and provide more robust evidence for the relationships observed.

Despite these limitations, our data could inform future research and potentially guide the development of targeted interventions aimed at supporting mental health and quality of life for women in this stage of life. Understanding and addressing the psychological aspects of menopause is essential for promoting healthy aging and fostering societal well-being.

## Data Availability

The data that support the findings of this study are available from the corresponding author, [G.O.], upon reasonable request.
